# Phase 2 and Later of COVID-19 Lockdown: Is it Possible to Perform Remote Diagnosis and Intervention for Autism Spectrum Disorder? An Online-Mediated Approach

**DOI:** 10.3390/jcm9061850

**Published:** 2020-06-13

**Authors:** Antonio Narzisi

**Affiliations:** IRCCS Stella Maris Foundation, 56018 Pisa, Italy; antonio.narzisi@fsm.unipi.it

**Keywords:** autism, COVID-19, children, diagnosis, intervention, parents, remote

## Abstract

COVID-19 is still in phase 2. The lockdown has been significantly reduced compared to phase 1. The centers and institutions that deal with the diagnosis and intervention of children with autism spectrum disorder (ASD) require rapid functional adaptation to respond to patients’ needs. The possibility of using technology to activate and manage diagnostic (preliminary diagnosis) and intervention processes should be explored. Two developed telemedicine working models for diagnosis and intervention, including synchronous and asynchronous transmissions, are presented. They are proposals not yet supported by the data. The diagnosis step is composed by two different and consecutives phases: (A) pre-specialistic consultation (PSC) and (B) specialistic assessment. The intervention step implemented well-recognized evidence-based models for preschoolers, school-aged, and older children in an online format. Parents’ support is also included. The described working models have the purpose of carrying out preliminary specialistic answers to the families without aiming to replace preferable in-person assessment. Based on previous research findings, the telemedicine approach is accepted by parents, increases their sense of competence, increases the parent intervention adhesion, and improves the social communication competencies for children with ASD. In conclusion, the presented working models must be considered partial responses to the current emergency status and at the same time as possible integrations into traditional approaches.

## 1. Introduction

COVID-19 is still in phase 2. The lockdown has been significantly reduced compared to phase 1. However, extreme attention and caution must be paid when carrying out daily activities. Gatherings must be avoided, and the safety distance must be maintained. For this reason, it is necessary to partially reorganize workplaces, where the presence of many people could represent a risk of contagion. Among these workplaces, the centers and institutions that deal with the diagnosis and intervention of children require rapid functional adaptation. Traditional methods for carrying out diagnostic assessments and intervention sessions will be able to resume at full capacity in phase 3 and later. So, in this bridging phase, we must think of new and agile ways to give at least an initial response to the clinical needs of families and patients [1]. Among the infant disorders, it is well recognized that autism spectrum disorder (ASD) has an elevated incidence, higher than 1/100 [2,3], and the requests for evaluation are many. Usually, the diagnosis of ASD involves a very close contact between the specialist and the children, especially when they are at preschool age. It could also be difficult and/or counterproductive to invite a child with ASD to wear certain safety devices (e.g., masks) during the diagnostic assessments. For this type of reason, it is necessary to think of a functional alternative between postponing the evaluation (in phase 3 or later) and/or making the child, family, and specialists run a high risk of contagion. At this time, the possibility of using technology to activate and manage diagnostic (or better, a preliminary diagnosis) and intervention processes in the field of ASD should be explored and tested.

In the field of ASD diagnosis, few studies have deepened the use of telemedicine. However, previous studies confirm the practicability, accuracy, and clinical efficacy of the telemedicine-based assessment of ASD for preschoolers and school-aged children [4,5]. Recent research contributions have shown the parents’ ability to gather clinically relevant videos of child behavior in the home setting and to share significant developmental history information, as well as the diagnosticians’ skill to detect appropriate behavioral examples in the videos to meet the diagnostic criteria for ASD [6,7].

Recently, Sutantio and colleagues [8] showed that a telemedicine methodology using a protocol-guided video recording evaluation has significant validity compared with direct assessment (DA) for diagnosing ASD.

In a pilot work, Juarez and colleagues [4] showed that a large portion (75%) of children with ASD may be accurately identified through the remote adoption of standardized assessment practices (reaching a sensitivity of 78.95%), and many parents and providers recognized the clinical value of the practice.

In a fascinating study, Fusaro and colleagues [9] tried to enlarge the concept and practicability of home video analysis by applying an Autism Diagnostic Observation Schedule (ADOS) [10] item, not the complete assessment, to ASD. Particularly, they tested the practicability of answering the ADOS module 1 item when viewing brief (10 min) formless videos to discriminate videos including children with ASD from videos of children who have no signs of ASD [9]. The results showed high classification accuracy (96.8% with 94.1% sensitivity and 100% specificity) and inter-rater reliability (88%) and together demonstrate that the ADOS module 1 item can be used on formless videos to effectively distinguish behavioral differences among children with and without ASD. Although not all items on the ADOS were expected to be pertinent to the formless videos, authors did find that most of the items could be applied. Items regarding vocalization, the use of words or phrases, unusual eye contact, responsive social smile, and repetitive interests or behaviors were the most recurrent behaviors shown in analyzed videos [9]. In conclusion, the authors demonstrated the potential for the video-based detection of ASD applying standard diagnostic items to ASD in short, formless home videos and further suggested that at least a portion of the effort associated with the detection and monitoring of ASD may be mobilized and moved outside of traditional clinical settings [9].

Unlike diagnosis, there is an increasing bulk of studies supporting the usefulness of telemedicine for intervention [11,12]. In 2018, Bearss and colleagues [13] carried out a feasibility pilot study of parent training with preschoolers with ASD using a telemedicine approach. The findings of their study were very promising; in fact, 93% of parents completed intervention, with almost 100% of sessions frequented (91.6%). Therapists reached 98% fidelity to the intervention guidelines and 93% of expected outcome measures were collected. Furthermore, 78.6% of children were evaluated as much/very much improved. Parent training through telemedicine was suitable to parents and the intervention could be carried out reliably by therapists. Among the studies in this area, it is important to report the randomized trial that compared telemedicine parent training in the Early Start Denver Model (P-ESDM) with a community intervention. Telemedicine training facilitated higher parent fidelity gains and program satisfaction for more of the P-ESDM than the community group at the end of the 12-week training period and at follow-up. The children’s social communication skills improved for both groups regardless of parent fidelity [14]. Even if results of this type need to be further studied, deepened, and better understood, they seem to recommend the feasibility of telemedicine training with an enriched parent intervention procedure and satisfaction from the program.

The main aim of this paper is to share telemedicine working models for preliminary diagnosis and intervention that we started to adopt at CETRA. CETRA is a highly specialized center for autism spectrum disorders (accredited by the Italian National Health Service) sited in Pisa, Italy. It offers in-person diagnosis and clinical intervention services, including evidence-based parent-mediated intervention. The in-person working model of CETRA involves the use of video feedback with parents during the intervention (inspired by the PACT model of Green and colleagues [15]) and the analysis of the videos used during the diagnostic process. Additional clinical offerings include support groups for parents, training for therapists, and research both in the area of diagnosis and intervention. Since the beginning of the COVID-19 period, most of the clinical activities have been remotely reorganized, developing a working model in telemedicine.

## 2. Material and Methods

### 2.1. A Telemedicine Working Model for Diagnosis

**Figure 1:**
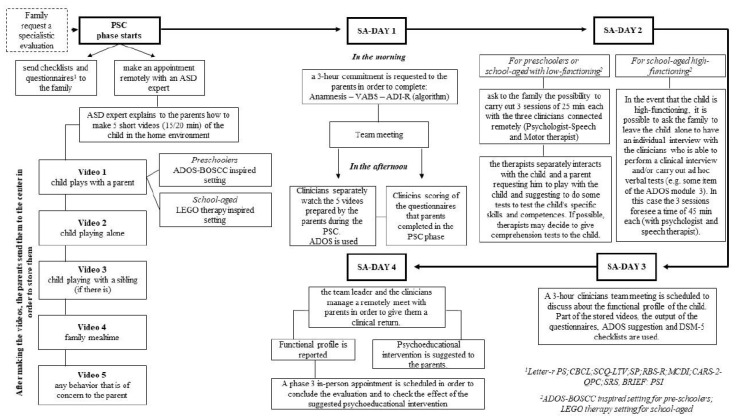
Working model for (preliminary) diagnosis. PSC: pre-specialistic consultation; SA: specialistic assessment; ASD: Autism Spectrum Disorder; ADOS-BOSCC: Autism Diagnostic Observation Schedule-Brief Observation of Social Communication Change; VABS: Vineland Adaptive Behaviour Scales; ADI-R: Autism Diagnostic Interview-Revised; DSM-5: Diagnostic and Statistical Manual of Mental Disorders-5.

The developed telemedicine working model for diagnosis is composed of two different and consecutives phases: (A) pre-specialistic consultation (PSC) and (B) specialistic assessment (SA) (see Figure 1).

#### 2.1.1. Pre-Specialistic Consultation (PSC)

The pre-specialistic consultation (PSC) phase starts when the family calls the reservation center to request a specialist evaluation. The receptionist, after having recorded the patient and family data, (1) sends the 11 questionnaires and checklists below to the family and (2) makes an appointment remotely (online Zoom platform or similar) with an expert psychologist in the field of the diagnosis of ASD.

#### 2.1.2. Questionnaire and Checklists

We use 11 questionnaires and checklists of which we have consolidated expertise:

(1) The Leiter–R parent social–emotional rating scales [16] provides the parent’s perception of the child’s cognitive/social functioning and emotion regulation.

(2) The Behavior Rating Inventory of Executive Function Preschool Version (BRIEF-P) [17] offers the possibility of a structured assessment of the executive functioning at preschool age, maximizing the opportunity to detect deficiencies and difficulties and to intervene promptly.

(3) The Behavior Rating Inventory of Executive Function 2, (BRIEF-2) [18] is a set of questionnaires for parents of school-aged children designed to evaluate executive function from multiple perspectives.

(4) The Child Behavior Check List (CBCL) [19] is a parent-report measure designed to record the problem behaviors of boys and girls. Each item describes a specific behavior and the parent is asked to rate its frequency on a three-point Likert scale. The scoring gives a summary profile including a DSM-oriented scale.

(5) The MacArthur Communicative Development Inventory (MCDI) [20] includes word comprehension, word expression, and gestures. Because the children could be older than those in the normative groups, raw data will be used instead of standard scores.

(6) The Questionnaire for Parents or Caregivers (CARS2-QPC) [21] is a parent report measure; the areas covered by the CARS2-QPC include the individual’s early development, social, emotional, and communication skills, repetitive behaviors, play and routines, and unusual sensory interests.

(7) The Repetitive Behavior Scale—Revised (RBS-r) [22] is a developed questionnaire that captures the following factors of RRB: ritualistic/sameness behavior, stereotypic behavior, self-injurious behavior, compulsive behavior, and restricted interests.

(8) The Social Communication Questionnaire (SCQ)–Life Time Form (SCQ-LT) [23] is filled out by parents to evaluate children’s communication, social, and relational skills. SCQ has established high comparative agreement with the Autism Diagnostic Interview—Revised™. The Lifetime Form focuses on the child’s entire developmental history.

(9) The Sensory Profile (SP) [24] evaluates the child’s sensory processing patterns in the context of home, school, and community-based activities. Parents indicate their perception of the frequency with which their child exhibits atypical behaviors in response to sensory stimulation. The SP evaluates tactile, visual/auditory, taste/smell, and movement sensitivity, auditory filtering, low energy/weakness, and sensation seeking [25].

(10) The Social Responsiveness Scale (SRS) [26] is a quick scale of evaluation of mutual social behavior, communication, and repetitive and stereotyped behaviors characteristic of autism spectrum disorders in children between four and 18 years of age.

(11) The Parenting Stress Index 4 [27] is focused on the clinical identification of specific problems and strengths in relation to the child, the parent, and the family system.

#### 2.1.3. Meeting with an Expert Psychologist in the Field of the Diagnosis of ASD

During the remote meeting the psychologist explains to the parents how to make some short videos of the child in the home environment.

At the end of the meeting, the psychologist sends to the family a brochure summarizing all the explained procedures to make videos. The PSC phase is distinguished by the age group of the children.

For preschoolers, the psychologist advises a parent to produce 15–20 min of the following five videos:

(a) The child playing with a parent (ADOS-BOSCC inspired setting [28]), 15 min.

A brief child–parent free play interaction aimed at observing the behavioral profile of the child. Parents are asked to place toys on the floor. Parents are instructed to play as usual, without making any additional demands on their child.

(b) The child playing alone (ADOS-2 free play inspired setting [10]), 15 min.

Parents are asked to place toys on the floor and on a small table (or a shelf). The request made to parents is to video record the child while he/she is playing with the toys that he/she spontaneously chooses from those proposed. The goal of this video is to observe the functional and symbolic use of objects, the sensory characteristics, the presence of restricted and repetitive behaviors, the presence of gergophasia, and the child’s ability to vary the play activity.

(c) The child playing with a sibling (if there is one), 15 min.

The (b) and (c) scenarios provide opportunities for the child to show typical social interaction skills and play. The setting is the same as video (b). In this case, the goal of the video is to observe the quality of social interaction with another child (in this case familiar). We are interested in observing social openings, the response to shared attention, the response to names, the child’s ability to draw attention, the quality of the social response, and the verbal and non-verbal communication skills.

(d) Family mealtime, 15 min.

The preferred setting for this video is that of breakfast, lunch, snack time, or dinner. The goal of this video is to observe the oral–motor skills and the presence of food selectivity [29]. This type of observation is very important both from the behavioral point of view and for ad hoc suggestions from the speech therapist.

(e) Any behavior that worries parents, 15 min;

The setting of this video is not minimally structured. The goal is to obtain a video recording that describes a specific behavior that has attracted the parents’ concern.

The points from (b) to (e) are inspired by Nazneen’s work [6,7].

For school-aged children, the videos and their duration are the same, except with regard video (a).

For these boys and girls, for video (a), it was decided to record their interaction with a parent in a setting similar to that of LEGO therapy [30]. The parent is asked to build a LEGO set (or other construction, or another game) together with the child. Through this, the therapist can remotely observe the child’s social skills, such as turn taking, problem solving, collaboration, and social communication.

For both preschoolers and school-aged children, all videos should have been made on different days in order to have a wider view of the child’s behavior.

The five videos and the questionnaires/checklists were sent via internet to the Diagnostic Autism Team at least 1 week before the start of phase B (specialistic assessment).

In the PSC phase, the family can request technical help from the psychologist at any time if doubts or uncertainties arise about how to prepare the videos. Overall, about 3 h were scheduled to complete the PSC phase.

### 2.2. Specialistic Assessment (SA)

The specialistic assessment phase requires a four-day commitment from the family.

During the first day, a 3 h commitment is requested from the parents (preferably in the morning). During this time, the psychologist carries out the anamnesis of the child (1 h) and administers the ADI-R algorithm [31] (1 h) and the Vineland/VABS [32,33,34] to the parent (1 h).

The Autism Diagnostic Interview—Revised (ADI-R) [31] is an interview aimed at obtaining a complete range of information for assessing autism spectrum disorders.

ADI-R is aimed at parents or educators of subjects from early childhood to adulthood with a mental age above 2 years. It focuses on the systematic and standardized observation of behaviors that are rarely found in non-clinical subjects, and mainly on the three areas of functioning: (1) language and communication, (2) mutual social interaction, and (3) stereotyped behaviors and restricted interests. The ADI-R is divided into an interview protocol and five algorithms, which can be used at various ages for diagnosis or intervention.

The Vineland Adaptive Behavior Scale—II Edition (VABS-II) [32] is a parent interview; it assesses adaptive behavior (AB). Specifically, VABS-II has the aim of measuring the AB in the domains of communication, daily living skills, socialization, and motor skills. The evaluation of AB is necessary for the diagnosis of intellectual disability disorder and, in accordance with the DSM-5, for the evaluation of the severity level of autistic disorder.

After anamnesis, ADI-R, and VABS-II administration, a team meeting (1 h) is scheduled to update everyone on the information received from the psychologist during the online meeting with the parents. After the meeting, the team, separately, watches the five videos prepared by the parents during the PSC phase and they finalize the scoring of the questionnaires that the parents completed in the PSC phase.

All clinicians watch the videos, although the global clinical evaluation of the five videos is performed by an expert clinical practitioner that is ADOS-2 certified, both for clinical and research use [10].

The ADOS-2 is widely considered a gold standard and is one of the most common behavioral instruments used to aid in the diagnosis of ASD [10]. It allows a semi-structured and standardized assessment of communication, social interaction, play, and restricted and repetitive behaviors, through a series of activities that directly elicit behaviors related to a diagnosis of autism spectrum disorder. Through the observation and coding of these behaviors, it is possible to obtain useful information for diagnosis, intervention planning, and insertion in educational contexts. The diagnostic algorithm consists of two domains, social affect and restricted, repetitive behaviors, combined into one score to which thresholds are applied [3,35]. As suggested by Fusaro and colleagues [9], the modules of the ADOS are used for the global clinical scoring of the five videos.

Following Fusaro and colleague’s indications, the code is applied if the video resolutely depicts a behavior and/or contains opportunities for the child to show the inquired behavior, otherwise the behavioral item is coded as not applicable (N/A) [9]. We calculate the ADOS-2 algorithm and then the videos are marked ASD when the score is > 7 (for module 1) and > 8 (for module 2). The ADOS modules 3 and 4 are administered in a remote connection with the patient and not in the analysis of asynchronous videos [36].

During the second day we ask to the family about the possibility of carrying out three remote sessions of 25 min each with the three clinicians. In the sessions, the clinicians interact with the child and a parent.

If the child is preschool aged, we ask the parent, in advance, to prepare a setting that is ADOS-BOSCC inspired [28]. If instead the child is older and not autonomous, we ask the parent to interact with him/her in a LEGO therapy-inspired setting [30].

In the three sessions, a psychologist, a speech therapist, and a psychomotor specialist interact, separately, with the parent, requesting him/her to play with the child and suggest some tests to check the child’s specific skills and competences. If possible, the clinicians may decide to give comprehension tests to the child. In the event that the child is high-functioning, it is possible to ask the family to leave the child alone to have an individual interview with the clinicians, who are able to perform a clinical interview and/or carry out ad hoc verbal tests (e.g., some items of ADOS module 3). In this case, the three sessions last for 45 min each (with psychologist and speech therapist separately).

During the third day, a 3 h team meeting is scheduled to discuss the functional profile of the child. Part of the stored videos and the output of the questionnaires and checklists are used. A checklist with Diagnostic and Statistical Manual (DSM-5) [37] criteria is used to help diagnose ASD. The family remains available for any questions from the therapists. The DSM-5 checklist includes a symptomatic dyad: (A) persistent deficits in social communication and social interaction across multiple contexts and (B) restricted, repetitive patterns of behavior, interests, or activities, which are made up of specific sub-criteria. Each of these categories includes from three to four sub-criteria. Criterion A is further divided into three sub-criteria: A1 (problems with social initiation and response), A2 (problems with nonverbal communication), and A3 (problems with social awareness and insight, as well as with the broader concept of social relationships). Criterion B is divided into four sub-criteria, including B1 (atypical speech, movements, and play), B2 (rituals and resistance to change), B3 (preoccupations with objects or topics) and B4 (atypical sensory behaviors). The DSM-5 contains specific examples and symptoms for each point [8,37].

The final preliminary diagnosis (ASD or non-ASD) is based on clinical judgement supported by the DSM-5 total checklist score, parent interviews, questionnaires/checklists, and through the analysis of both the five videos recorded by the parents (asynchronous transmission) on which the ADOS module is applied by an experienced psychologist and the direct observation of the child in live video conferencing (synchronous transmission).

During the fourth day, the team leader, together with clinicians, manage a remote meeting with the parents in order to give them clinical feedback about the functional profile and the preliminary diagnosis of their child. During the meeting, individualized psychoeducational advice is provided to parents. Overall, about 13 h are scheduled to complete the SA phase (9 h for evaluations and 4 h for meetings).

## 3. A Telemedicine Working Model for Intervention

**Figure 2:**
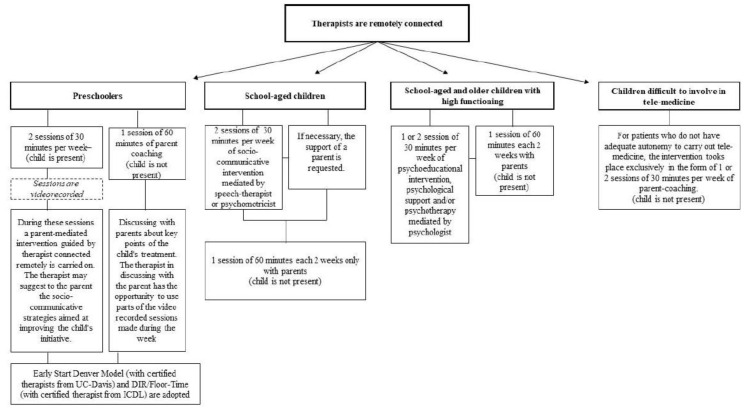
Working model for intervention.

The telemedicine working model for intervention that has been developed was aimed at both preschoolers and older children (see Figure 2).

For preschoolers, two sessions of 30 min each per week are expected. During these sessions, a parent-mediated remote intervention, guided by therapist, is carried out. The therapist may suggest to the parents the socio-communicative strategies aimed at improving the child’s initiative. The two sessions (with parent–child and therapist remotely connected) are video recorded. During the week, another session of 60 min (remotely) is added to the two described before with the purpose of discussing with the parents the key points of the child’s intervention (without the child). The therapist, in the discussion with the parents, can use parts of the recorded sessions made during the week.

For the preschoolers, the adopted intervention models are the so-called Naturalistic Developmental Behavioral Interventions (NDBI) [38]. The NDBI are evidence-based intervention models; they are based both on behavioral learning and on developmental sciences. The Early Start Denver Model [39,40] (with certified therapists from University of California (UC)-Davis) and DIR/Floor-Time [41] (with certified therapist from Interdisciplinary Council on Development and Learning (ICDL)) are remotely implemented. Remote parenting coaching, to provide parents/caregivers with tools and strategies to teach and engage their child through play and everyday routines, such as mealtimes, bathing, and dressing, is provided [42].

Therapists are supervised by an experienced psychologist in the field of early intervention.

For school-aged children, two 30 min online speech and communication therapy sessions are suggested per week. If necessary, the support of a parent is requested. The efficacy of speech and communication therapy delivered via telemedicine has been well documented [43]. During speech and communication therapy, the therapist and child are remotely connected, and they can interact in real time through audio and video with images and learning materials [43]. One session of 60 min every two weeks with parents is provided (without the child) to share the child’s improvements and difficulties, as well as to suggest practical advice for the parents to perform at home.

For high-functioning children, it is established to continue the psychoeducational interventions, psychological support, and psychotherapy; however, the duration was modified to 30 min a week instead the original 60 min of in-person therapy. The high psychiatric vulnerability and/or comorbidity of children with ASD is widely documented. Among them, anxiety disorder is one of the most reported [44]. Psychiatric comorbidities could contribute to a depletion of development, especially in adolescence. The alert state caused by the COVID-19 pandemic could be a difficult event to mentalize for children with ASD. For this reason, if the children were engaged in psychotherapy before the COVID-19 alert, it is important that they continue the therapy in online video or audio mode with the same weekly appointments. Continuing therapy could reduce anxiety, control mood, and offer children a private space to talk to a specialist [1].

One session of 60 min every two weeks with parents is also provided (without the child).

For patients who do not have the adequate autonomy to carry out online therapy, the intervention takes place exclusively in the form of one or two 30 min sessions a week for remote parent coaching.

During remote parent coaching, parents are invited to (1) share a home video (10–15 min) with therapists related to the child’s behavior during structured sessions in a home setting or during free play and (2) discuss with therapists individualized strategies and methods of intervention for their child.

## 4. Conclusions

Our telemedicine working models represent a union between synchronous transmission (i.e., live video conferencing as two-way video and audio interactions between the therapists and parents/child for interviews and other behavioral observation of the child) and asynchronous transmission (i.e., “store and forward” transmissions of recorded video, data, and questionnaires/checklists) [45].

Telemedicine should not totally take the place of in-person clinical services; however, in this moment, it could be required to provide answers to families who are concerned about the health of their children with ASD. The described working model for diagnosis has the purpose of carrying out a preliminary assessment which does not have the purpose of replacing preferable in-person assessment. The described working model for diagnosis may not be suitable for all families, and in some cases, it could be even contraindicated (i.e., low confidence with technology). For some of them, it is difficult to perform a remote evaluation. Therefore, in some cases, the in-person evaluation cannot be replaced even partially by the remote one. However, for other children, this working model for diagnosis can provide useful indications and suggest the start of an individualized intervention. It is our decision that all the children who carry out the remote evaluation with telemedicine have an in-person appointment when phase 3 of the lockdown begins, in order to conclude the evaluation and to check the effect of the suggested psychoeducational intervention. The presented working model represents a scheme which obviously can be flexible; therefore, it is possible, in some cases, to increase the number of expected days to complete the evaluation. The presented working model for diagnosis could permit clinicians to look at otherwise inaccessible children’s behaviors in their natural setting, and to observe parent–child reciprocity [7]. The proposed working model for diagnosis makes it possible for parents to register videos in their home, during their daily activities, which permits the capture of natural expressions of child behavior that are broadly recognized as essential to a precise and comprehensive assessment [6]. The background of the presented working model for diagnosis is based on previous studies that showed that a telemedicine procedure could be adequate for acquiring children’s diagnostic profiles in a way that parents report as easy and acceptable [4]. Currently, the clinical judgment of the professional must establish the adequacy of telemedicine on a case-by-case basis.

The described working model for intervention with ASD children is not exhaustive and it needs to be tried out; however, it is supported from preliminary research findings in this field [14,46]. The telemedicine approach is accepted by parents [13], increases their sense of competence [47], increases the parent intervention accuracy, and improves the socio-communicative competencies of children with ASD [41,48].

Because of distancing and lockdowns, a functional use of telemedicine is pivotal. Clinical services are moving to digitalization and remote approaches to respond current patients’ needs [49,50]. During COVID-19 phase 2 and later, telemedicine and in-person diagnosis and intervention must alternate.

In the future, even when the COVID-19 alert expires, telemedicine can play an essential role in speeding up the autism diagnosis process. Because of the high number of evaluation requests for ASD, there are very long waiting lists. The systematic increase in telemedicine, together with traditional assessment in-person procedures will significantly decrease the needed times for a diagnostic indication. Early diagnosis is crucial to help a child with ASD, since early identification can significantly improve the child’s developmental trajectory. Moreover, research has shown that young children who receive the early intervention can have higher chances of demonstrating significant gains in functioning than children diagnosed later.

In conclusion, the presented working models for preliminary diagnosis and intervention must be considered as both a partial response to the current emergency status and, at the same time, as a possible integration into traditional approaches.

## References

[1] Narzisi A. (2020). Handle the Autism Spectrum Condition during Coronavirus (COVID-19) Stay at Home period: Ten Tips for Helping Parents and Caregivers of Young Children. Brain Sci..

[2] Maenner M.J., Shaw K.A., Baio J., Washington A., Patrick M., DiRienzo M., Christensen D.L., Wiggins L.D., Pettygrove S., Andrews J.G. (2020). Prevalence of Autism Spectrum Disorder Among Children Aged 8 Years—Autism and Developmental Disabilities Monitoring Network, 11 Sites, United States, 2016. Mmwr Surveill. Summ..

[3] Narzisi A., Posada M., Barbieri F., Chericoni N., Ciuffolini D., Pinzino M., Romano R., Scattoni M.L., Tancredi R., Calderoni S. (2018). Prevalence of Autism Spectrum Disorder in a large Italian catchment area, A school-based population study within the ASDEU project. Epidemiol. Psychiatr. Sci..

[4] Juárez A.P., Weitlauf A.S., Nicholson A., Pasternak A., Broderick N., Hine J., Stainbook J.A., Warren Z. (2018). Early Identification of ASD through Telemedicine: Potential Value for Underserved Populations. J. Autism Dev. Disord..

[5] Sutherland R., Trembath D., Hodge M.A., Rose V., Roberts J. (2019). Telehealth and autism: Are telehealth language assessments reliable and feasible for children with autism?. Int. J. Lang. Commun. Disord..

[6] Smith C., Rozga A., Matthews N., Oberleitner R., Nazneen N., Abowd G. (2017). Investigating the accuracy of a novel telehealth diagnostic approach for Autism Spectrum Disorder. Psychol Assess..

[7] Nazneen N., Rozga A., Smith C., Oberleitner R., Abowd G., Arriaga R. (2015). A Novel System for Supporting Autism Diagnosis Using Home Videos: Iterative Development and Evaluation of System Design. J. Mir Mhealth Uhealth..

[8] Sutantio J.D., Pusponegoro H.D., Sekartini R. (2020). Validity of Telemedicine for Diagnosing Autism Spectrum Disorder: Protocol-Guided Video Recording Evaluation. Telemed. E-Health.

[9] Fusaro V.A., Daniels J., Duda M., DeLuca T.F., D’Angelo O., Tamburello J., Maniscalco J., Wall D.P. (2014). The Potential of Accelerating Early Detection of Autism through Content Analysis of YouTube Videos. PLoS ONE.

[10] Lord C., Luyster R., Gotham K., Guthrie W. (2012). (ADOS-2) Manual (Part II): Toddler Module. Autism Diagnostic Observation Schedule.

[11] Johnsson G., Kerslake R., Crook S. (2019). Delivering allied health services to regional and remote participants on the autism spectrum via video-conferencing technology: Lessons learned. Rural Remote Health..

[12] Carter A.S., Messinger D.S., Stone W.L., Celimli S., Nahmias A.S., Yoder P. (2011). Randomized controlled trial of Hanen’s ‘More Than Words’ in toddlers with early autism symptoms. Child Psychol. Psychiatry.

[13] Bearss K., Burrell T.L., Challa S.A., Postorino V., Gillespie S.E., Crooks C., Scahill L. (2018). Feasibility of Parent Training via Telehealth for Children with Autism Spectrum Disorder and Disruptive Behavior: A Demonstration Pilot. J. Autism Dev. Disord..

[14] Vismara L., McCormick C., Wagner A., Monlux K., Nadhan A., Young G. (2018). Telehealth Parent Training in the Early Start Denver Model: Results From a Randomized Controlled Study. Focus Autism Other Dev. Disabil..

[15] Green J., Charman T., McConachie H., Aldred C., Slonims V., Howlin P., Le Couteur A., Leadbitter K., Hudry K., Byford S. (2010). Parent-mediated communication-focused treatment in children with autism (PACT): A randomised controlled trial. Lancet.

[16] Roid G.L., Roid G.H., Miller L.J. (1997). Leiter international performance scale-revised: Examiner’s manual. Leiter International Performance Scale-Revised.

[17] Greene J.A., Trujillo S., Isquith P.K., Gioia G.A., Espy K.A. (2019). Enhanced Interpretation of the Behavior Rating Inventory of Executive Function–Preschool Version (BRIEF-P) (White Paper).

[18] Gioia G.A., Isquith P.K., Guy S.C., Kenworthy L. (2015). Behavior Rating Inventory of Executive Function® (BRIEF®2).

[19] Achenbach T.M., Rescorla L. (2000). Manual for the ASEBA Preschool Forms and Profile.

[20] Fenson L., Pethick S., Renda C., Cox J.L., Dale P.S., Reznick J.S. (2000). Short-form versions of the MacArthur Communicative Developmental Inventories. Appl. Psycholinguist..

[21] Schopler E., Van Bourgondien M.E., Wellman G.J., Love S.R. (2010). Childhood Autism Rating Scale.

[22] Lam K.S., Aman M.G. (2007). The Repetitive Behavior Scale-Revised: Independent validation in individuals with autism spectrum disorders. J. Autism Dev. Disord..

[23] Rutter M., Bailey A., Lord C. (2000). Social Communication Questionnaire (SCQ) Manual.

[24] Dunn W. (1999). Sensory Profile.

[25] James K., Lucy J., Millera L.J., Schaaf R., Nielsene D.M., Schoena S.A. (2011). Phenotypes within sensory modulation dysfunction. Compr. Psychiatry.

[26] Constantino J., Gruber J. (2005). Social Responsiveness Scale (SRS) Manual.

[27] Abidin R.R. (2012). Parenting Stress Index.

[28] Grzadzinski R., Carr T., Colombi C., McGuire K., Dufek S., Pickles A., Lord C. (2016). Measuring Changes in Social Communication Behaviors: Preliminary Development of the Brief Observation of Social Communication Change (BOSCC). J. Autism Dev. Disord..

[29] Ausderau K.K., St John B., Kwaterski K.N., Nieuwenhuis B., Bradley E. (2019). Parents’ Strategies to Support Mealtime Participation of Their Children With Autism Spectrum Disorder. Am. J. Occup. Ther..

[30] Peckett H., MacCallum F., Knibbs J. (2016). Maternal experience of Lego Therapy in families with children with autism spectrum conditions: What is the impact on family relationships?. Autism.

[31] Lord C., Rutter M., Le Couteur A. (1994). Autism Diagnostic Interview-Revised: A revised version of a diagnostic interview for caregivers of individuals with possible pervasive developmental disorders. J. Autism Dev. Disord..

[32] Sparrow S.S., Balla D.A., Cicchetti D.V., Doll E.D. (2005). Vineland-II: Vineland Adaptive Behavior Scales: Survey Forms Manual.

[33] Balboni G., Tasso A., Muratori F., Cubelli R. (2016). The Vineland-II in preschool children with Autism Spectrum Disorders: An item content category analysis. J. Autism Dev. Disord..

[34] Balboni G., Belacchi C., Bonichini S., Coscarelli A. (2016). Vineland-II. Vineland Adaptive Behavior Scales Second Edition Survey Interview Form.

[35] Gotham K., Risi S., Pickles A., Lord C. (2007). The Autism Diagnostic Observation Schedule: Revised Algorithms for Improved Diagnostic Validity. J. Autism Dev. Disord..

[36] Schutte J.L., McCue M.P., Parmanto B., McGonigle J., Handen B., Lewis A., Pulantara I.W., Saptono A. (2015). Usability and Reliability of a Remotely Administered Adult Autism Assessment, the Autism Diagnostic Observation Schedule (ADOS) Module 4. Telemed J. E Health.

[37] APA (2013). Diagnostic and Statistical Manual of Mental Disorders.

[38] Schreibman L., Dawson G., Stahmer A.C., Landa R., Rogers S.J., McGee G.G., Kasari C., Ingersoll B., Kaiser A.P., Bruinsma Y. (2015). Naturalistic Developmental Behavioral Interventions: Empirically Validated Treatments for Autism Spectrum Disorder. J. Autism Dev. Disord..

[39] Dawson G., Rogers S., Munson J., Smith M., Winter J., Greenson J., Donaldson A., Varley J. (2010). Randomized, controlled trial of an intervention for toddlers with autism: The Early Start Denver Model. Pediatrics.

[40] Colombi C., Narzisi A., Ruta L., Cigala V., Gagliano A., Pioggia G., Siracusano R., Rogers S.J., Muratori F., Prima Pietra Team (2018). Implementation of the Early Start Denver Model in an Italian community. Autism.

[41] Greenspan S.I., Wieder S., Simons R. (1998). The Child with Special Needs: Encouraging Intellectual and Emotional Growth.

[42] Rogers S.J., Dawson G., Vismara L.A. (2012). An Early Start for Your Child with Autism: Using Everyday Activities to Help Kids Connect, Communicate, and Learn.

[43] Towey M.P. (2012). Speech Telepractice: Installing a Speech Therapy Upgrade for the 21st Century. Int. J. Telerehabilitation.

[44] Lai M.C., Kassee C., Besney R., Bonato S., Hull L., Mandy W., Szatmari P., Ameis S.H. (2019). Prevalence of co-occurring mental health diagnoses in the autism population: A systematic review and meta-analysis. Lancet Psychiatry.

[45] CASP The Council of Autism. Organizational Guidelines and Standards.

[46] Parsons D., Cordier R., Vaz S., Lee H.C. (2017). Parent-Mediated Intervention Training Delivered Remotely for Children With Autism Spectrum Disorder Living Outside of Urban Areas: Systematic Review. J. Med. Internet Res..

[47] Hepburn S.L., Blakeley-Smith A., Wolff B., Reaven J.A. (2016). Telehealth delivery of cognitive-behavioral intervention to youth with autism spectrum disorder and anxiety: A pilot study. Autism.

[48] Ingersoll B., Berger N.I. (2015). Parent Engagement with a Telehealth-Based Parent-Mediated Intervention Program for Children with Autism Spectrum Disorders: Predictors of Program Use and Parent Outcomes. J. Med. Internet Res..

[49] Townsend E., Nielsen E., Allister R., Cassidy S.A. (2020). Key ethical questions for research during the COVID-19 pandemic. Lancet Psychiatry.

[50] Stainbrook J.A., Weitlauf A.S., Juárez A.P., Taylor J.L., Hine J., Broderick N., Nicholson A., Zachary W. (2019). Measuring the service system impact of a novel telediagnostic service program for young children with autism spectrum disorder. Autism.

